# Dysregulation of the Heparanase/TFPI-2/Heparan Sulfate Axis in Recurrent Pregnancy Loss

**DOI:** 10.3390/ijms27188210

**Published:** 2026-09-15

**Authors:** Chen Yanovich, Nadin Sabbah, Keren Asayag, Yonatan Crispel, Haim Cohen, Anat Keren-Politansky, Yona Nadir

**Affiliations:** 1 Thrombosis and Hemostasis Unit, Rambam Health Care Campus, Haifa 31096, Israel; 2 The Rappaport Faculty of Medicine, Technion—Israel Institute of Technology, Haifa 31096, Israel

**Keywords:** recurrent pregnancy loss, heparanase, tissue factor pathway inhibitor 2, heparan sulfate, thrombophilia

## Abstract

Recurrent pregnancy loss (RPL) is a clinically heterogeneous reproductive disorder, and in many women the underlying mechanism remains unexplained by standard thrombophilia evaluation. Pregnancy is characterized by a tightly regulated hemostatic balance at the maternal–placental interface. Heparanase, tissue factor pathway inhibitor 2 (TFPI-2), and heparan sulfate (HS) are expressed in placental and hemostatic pathways and may contribute to coagulation-related pregnancy complications. We investigated whether the heparanase/TFPI-2/HS axis is altered in women with RPL. Blood samples were obtained from 69 women with RPL and 47 control women with at least two normal deliveries and no history of pregnancy-related vascular complications. Samples were collected at least three months after pregnancy and in the absence of hormonal therapy. Plasma and white blood cell samples were analyzed using an enzyme-linked immunosorbent assay, Western blotting, real-time polymerase chain reaction, co-immunoprecipitation, and Sanger sequencing. Mechanistic experiments using heparanase-derived peptide 16 and TFPI-2-derived peptide 6 were performed in MCF-7 and JAR cells. We found that women with RPL had increased plasma heparanase levels, heparanase procoagulant activity, HS levels, and heparanase mRNA expression compared with controls. TFPI-2 protein and mRNA expression were also elevated. The heparanase rs4693608 AA genotype was more frequent in women with RPL and, together with rs4364254 TT, was associated with increased heparanase expression and procoagulant activity. Although TFPI-2 levels were higher in RPL, co-immunoprecipitation showed reduced heparanase/TFPI-2 complex formation, suggesting impaired inhibitory regulation. HS modulated heparanase binding to TF and TFPI-2 in a concentration-dependent, bell-shaped manner. Heparanase-derived peptide 16 increased TFPI-2 expression, supporting a regulatory feedback loop. In conclusion, RPL is associated with dysregulation of the heparanase/TFPI-2/HS axis, including increased heparanase expression and activity, altered heparanase/TFPI-2 interaction, and heparanase-related genetic variation. These findings suggest a coagulation-related reproductive mechanism that may contribute to pregnancy loss and warrants validation in larger prospective studies.

## 1. Introduction

Pregnancy is an acquired hypercoagulable state, and women with a prior tendency toward thrombosis may present with clinical manifestations of placental vascular complications. Maternal thrombophilia can be linked to placental vascular events, although a substantial proportion of vascular gestational pathologies cannot be explained by currently available thrombophilia tests [1,2,3,4,5,6]. Thus, the need to understand the delicate hemostatic balance and the dominant factors that regulate it throughout pregnancy is essential. In this context, heparanase, tissue factor pathway inhibitor 2 (TFPI-2), and heparan sulfate are plausible candidates because they are abundant in placental tissues and may influence both anticoagulant and procoagulant signaling. Notably, the normal function of the endothelium in the placenta is multifactorial and hemostasis is one aspect involved. Alternative endothelial and trophoblast adhesion and junction molecules play a critical role in regulating the structural integrity of the placental–maternal interface. Dysregulation of these alternative molecules leads to significant placental pathologies [7,8].

Heparanase, an enzyme that degrades heparan sulfate (HS) chains, is known to enhance inflammation, angiogenesis and metastasis [9,10]. We previously demonstrated that heparanase might also affect the hemostatic system in a non-enzymatic manner [11,12,13]. Our findings demonstrated that heparanase directly enhanced tissue factor (TF) activity, leading to increased factor Xa production and subsequent activation of the coagulation system [13]. Our earlier studies showed that heparanase upregulated the expression of TF [11] and interacted with the tissue factor pathway inhibitor (TFPI) on the cell surface membrane of endothelial and tumor cells, resulting in TFPI dissociation and increased cell surface coagulation activity [12]. In addition, it was shown that heparanase was found to induce vascular endothelial growth factor A (VEGF-A) expression [14,15]. Moreover, it was shown that heparanase plays an important role in the placenta hemostasis and its effect on TFPI-2 and VEGF-A in early pregnancy losses. Placentas from early pregnancy losses of woman with thrombophilia demonstrated high protein and mRNA levels of heparanase, TFPI-2 and VEGF-A compared with pregnancy terminations of women with normal obstetric history. Additionally, it was shown that heparanase upregulates both TFPI and TFPI-2 in trophoblasts [1]. These results indicate a regulatory effect of heparanase on these coagulation proteins and suggesting a potential involvement of heparanase in miscarriages. The main novelty of the present study is the evaluation of the circulating heparanase/TFPI-2/HS axis in women with RPL, extending our previous placental observations. Specifically, we examine the direct heparanase/TFPI-2 interaction, its modulation by HS and heparins, and the heparanase-dependent regulation of TFPI-2 expression.

## 2. Results

The study included 69 women with recurrent pregnancy loss (RPL) and 47 controls with at least two normal deliveries and no history of pregnancy vascular complications. Blood was collected at least three months after pregnancy and at least three months after hormonal therapy; plasma and peripheral white blood cell fractions were analyzed. Samples used for Western blotting and co-immunoprecipitation were randomly selected from the larger cohort as specified below. The principal study measurements were plasma heparanase level, heparanase procoagulant activity, heparan sulfate (HS), TFPI-2 protein, heparanase and TFPI-2 mRNA expression, and the heparanase SNPs rs4693608 and rs4364254. Thrombophilia testing was performed only in the RPL group and included lupus anticoagulant, anti-cardiolipin, anti-β2-glycoprotein, prothrombin mutation, factor V Leiden, factor VIII, homocysteine, protein C, protein S, and antithrombin. The mean age was 31 ± 5 years in the RPL group and 32 ± 4 years in controls, and the mean weight was 71 ± 18 and 67 ± 16, respectively; these parameters did not differ significantly between groups. Fifty-one patients (74%) had at least one thrombophilia marker, five (8%) had two markers, and 18 (26%) had a normal work-up. The thrombophilia findings are presented in Table 1.

**Figure 1:**
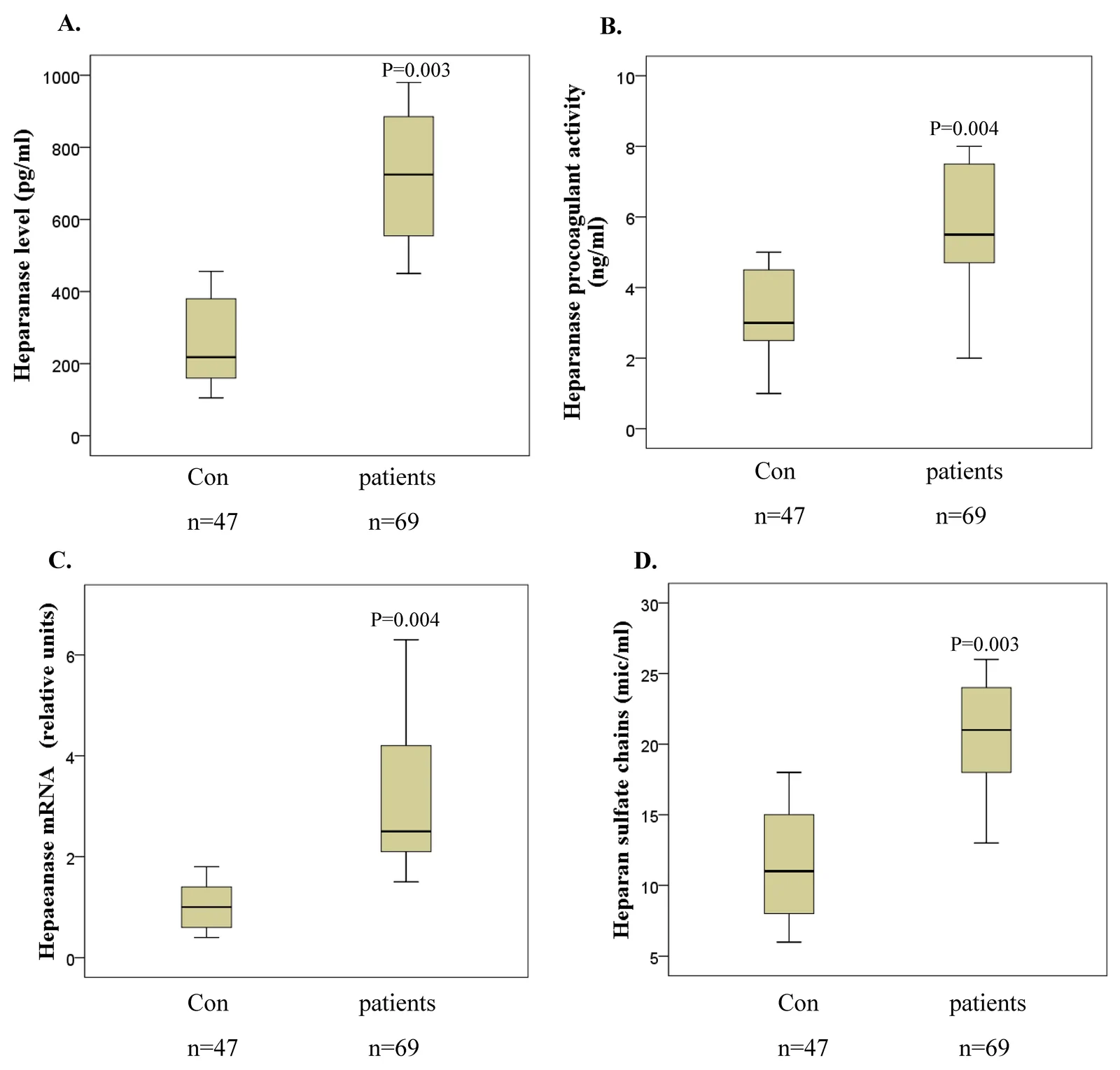

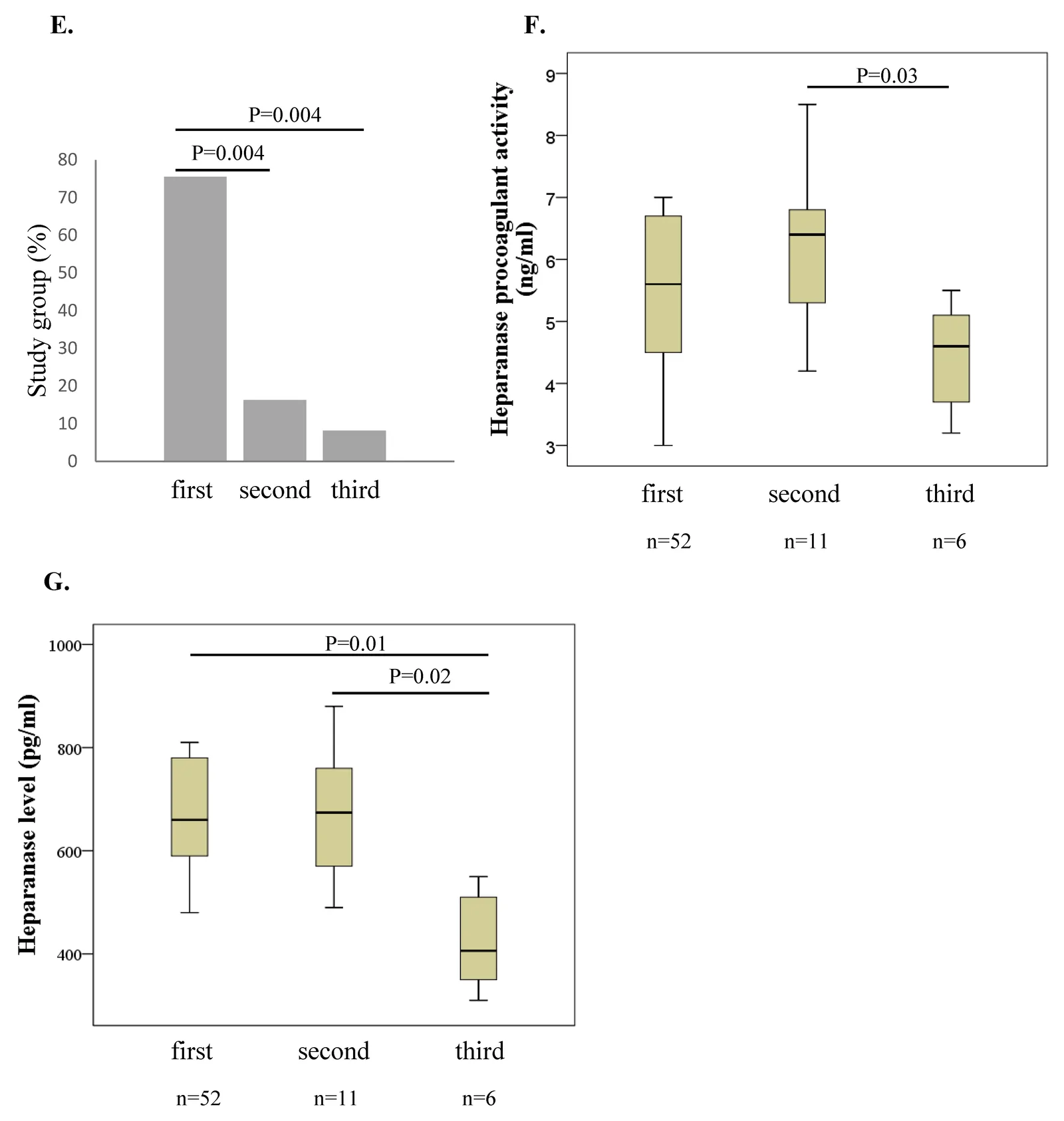

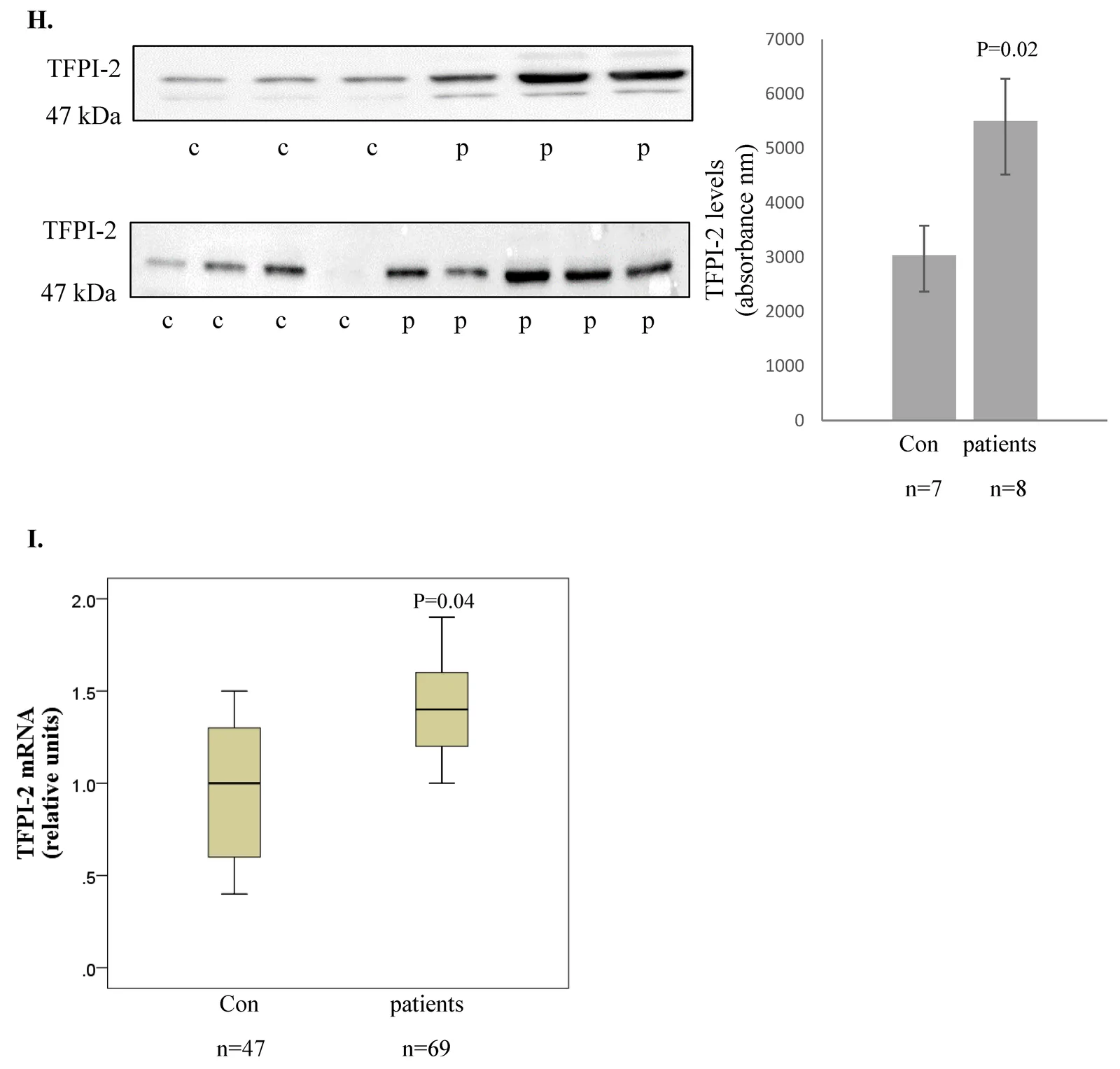
Measurements of heparanase, HS, and TFPI-2 in women with RPL and controls. (**A**) Plasma heparanase measured by ELISA; (**B**) heparanase procoagulant activity measured by factor Xa generation assay; (**C**) heparanase mRNA in peripheral WBCs measured by qRT-PCR; (**D**) plasma HS measured by chromogenic assay; n = 47 controls and n = 69 patients for panels A-D. (**E**) Distribution of patients according to the highest trimester of pregnancy loss (n = 52 first, n = 11 s, n = 6 third). (**F**,**G**) Heparanase procoagulant activity and plasma heparanase level stratified by trimester of loss. (**H**) Plasma TFPI-2 measured by Western blot and densitometry in controls (n = 7) and patients (n = 8), analyzed on two separate gels. (**I**) TFPI-2 mRNA in WBCs measured by qRT-PCR (n = 47 controls, n = 69 patients). Box plots show median, 25th–75th percentiles, and 10th–90th percentiles. Unpaired t test was used for panels A-G and I; Mann–Whitney U test was used for panel H. RPL—recurrent pregnancy loss; HS—heparan sulfate; TFPI-2—tissue factor pathway inhibitor 2; WBC—white blood cell; ELISA—enzyme-linked immunosorbent assay; qRT-PCR—quantitative real-time polymerase chain reaction.

Next, we compared heparanase and TFPI-2 measurements between women with RPL and controls. According to the exclusion criteria described in the Materials and Methods, blood samples were collected at least 3 months after pregnancy and at least 3 months after hormonal therapy. We measured plasma heparanase levels, heparanase procoagulant activity, and HS, a product of heparanase enzymatic activity, and assessed heparanase mRNA expression in peripheral white blood cells (Figure 1A–D). In all four assays, values were higher in women with RPL than in women with a normal obstetric history. We also analyzed the heparanase level and procoagulant activity according to the trimester of pregnancy loss, with patients classified by their highest trimester of loss. Most women experienced first-trimester losses (Figure 1E). Heparanase level and procoagulant activity were higher in the first- and second-trimester groups than in the third-trimester group; however, the numbers in the second- and third-trimester groups were small (Figure 1F,G). TFPI-2 protein was assessed independently by Western blot and densitometry in randomly selected plasma samples from women with pregnancy loss (n = 8) and controls (n = 7), analyzed on two separate gels (Figure 1H). TFPI-2 mRNA was assessed separately in peripheral white blood cells by qRT-PCR in the full cohort (Figure 1I). Unless otherwise indicated, assays were performed in triplicate, data are presented as median with 25–75th and 10–90th percentiles, and comparisons were made using an unpaired *t* test; TFPI-2 Western blot data were analyzed via a Mann–Whitney U test and are presented as the median and range.

**Figure 2:**
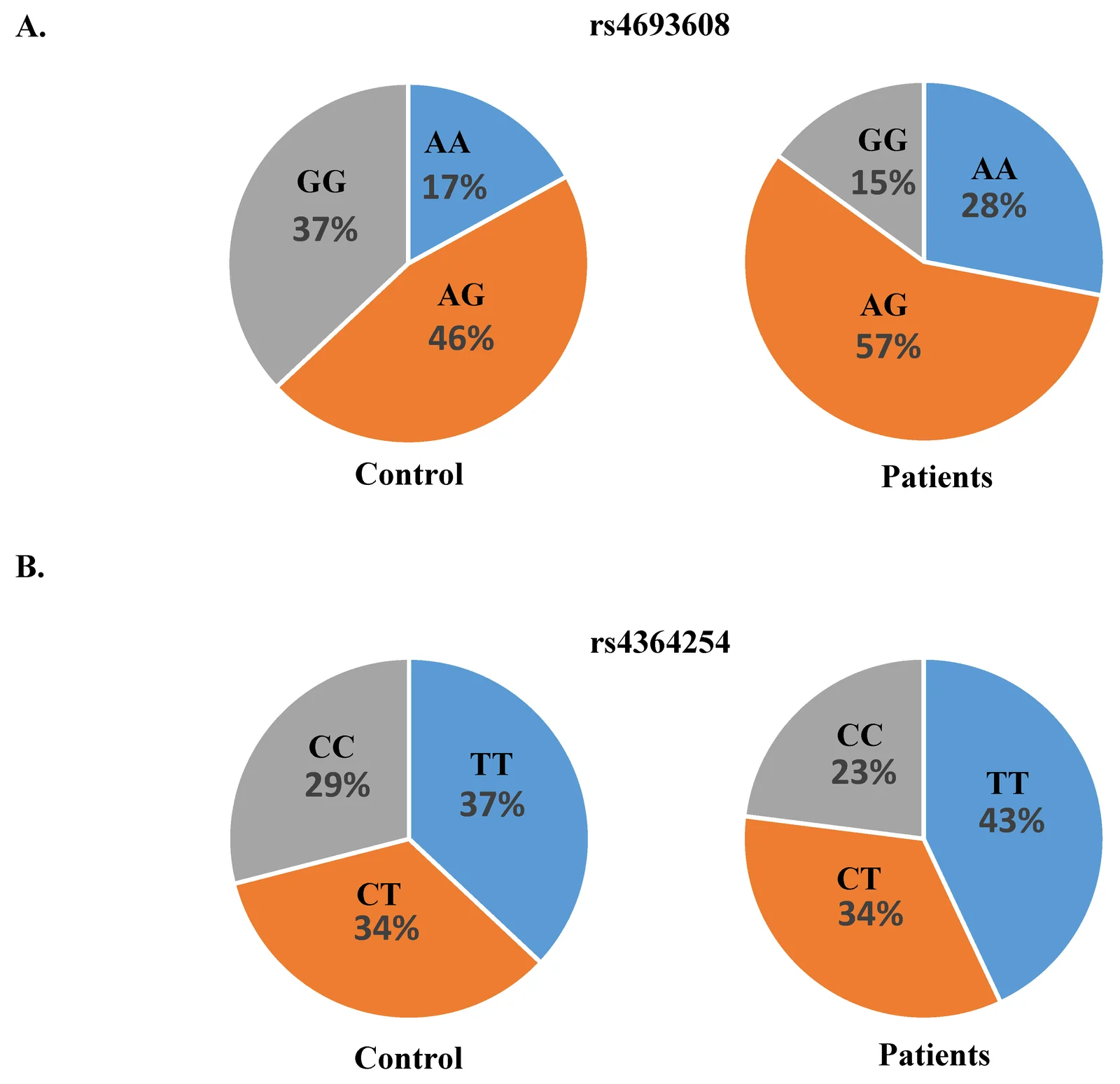

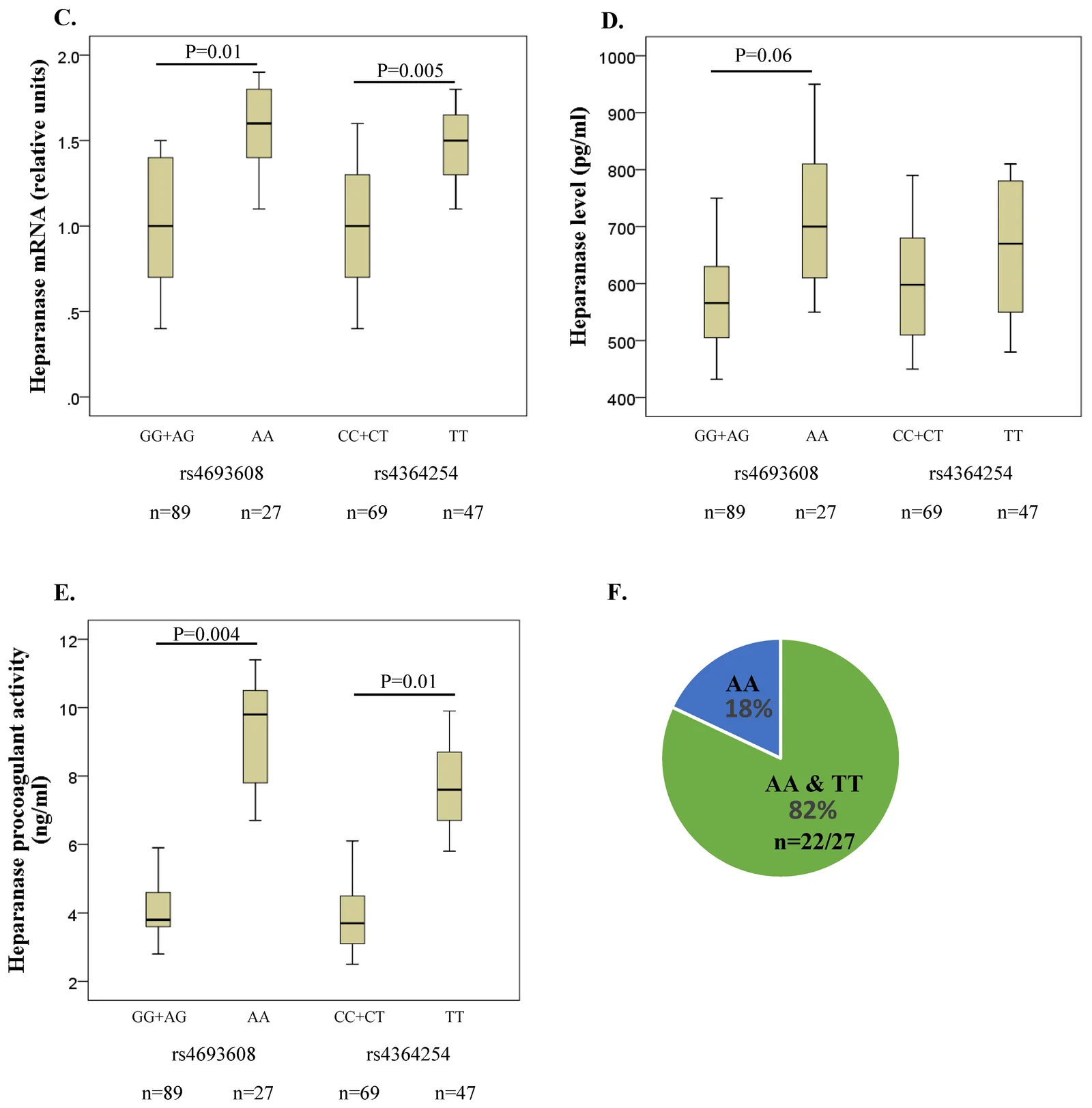
Heparanase SNP analysis in women with RPL and controls. rs4693608 (G > A) and rs4364254 (C > T) were genotyped by Sanger sequencing; complete paired genotype data used for the requested association analyses were available for 69 patients and 47 controls. (**A**,**B**) Genotype distributions for rs4693608 and rs4364254; overall genotype distributions were compared by a Chi-square test, and allele and dominant/recessive models were evaluated using Fisher’s exact tests. (**C**–**E**) Heparanase mRNA expression, plasma heparanase level, and heparanase procoagulant activity stratified by rs4693608 (GG + AG versus AA) and rs4364254 (CC + CT versus TT); unpaired t-test; n values are shown in the panels. (**F**) Concurrent rs4693608 AA and rs4364254 TT genotypes among AA carriers. Exploratory two-locus haplotypes were also estimated by an expectation-maximization procedure as described in the text. Box plots show median, 25th–75th percentiles, and 10–90th percentiles. SNP—single-nucleotide polymorphism; RPL—recurrent pregnancy loss.

Heparanase SNPs rs4693608 (G > A) and rs4364254 (C > T) were examined in women with RPL and controls. Previous work showed that combinations of these two SNPs stratify heparanase expression levels among healthy individuals [16]. Ostrovsky et al. identified a strong enhancer within intron 2 of heparanase and showed that rs4693608 influences enhancer activity; greater heparanase binding to the enhancer was observed with the G allele, consistent with stronger negative feedback and lower expression than with the AA genotype [16]. Complete paired genotype data for the two SNPs were available for 69 patients and 47 controls and were used for the additional genetic analyses requested by the reviewer. For rs4693608, genotype frequencies were AA 28%, AG 57%, and GG 15% in patients and AA 17%, AG 46%, and GG 37% in controls. The overall genotype distribution differed between groups (Chi-square = 6.50, *p* = 0.039). The A-allele frequency was 56.6% in patients and 40.0% in controls (OR = 1.95, 95% CI 1.07–3.55; Fisher exact *p* = 0.036). In an A-dominant model (AA + AG versus GG), the association remained significant (OR = 3.41, 95% CI 1.27–9.15; Fisher exact *p* = 0.022), whereas the A-recessive model (AA versus AG + GG) was not significant (*p* = 0.322). For rs4364254, genotype frequencies were TT 43%, CT 34%, and CC 23% in patients and TT 37%, CT 34%, and CC 29% in controls. The overall genotype distribution was not different between groups (Chi-square = 0.45, *p* = 0.800). The T-allele frequency was 59.8% in patients and 54.3% in controls (OR = 1.25, 95% CI 0.69–2.27; Fisher exact *p* = 0.544), and neither the T-dominant nor T-recessive model was significant (*p* = 0.626 and *p* = 0.669, respectively). Genotype distributions in controls were compatible with Hardy–Weinberg equilibrium for rs4693608 (*p* = 0.778) and rs4364254 (*p* = 0.067). Exploratory two-locus haplotype frequencies were estimated from the unphased genotypes by an expectation-maximization procedure. The estimated AC, AT, GC, and GT haplotype frequencies were 11.2%, 45.4%, 29.0%, and 14.5% in patients and 8.3%, 31.7%, 37.4%, and 22.6% in controls; the global haplotype-distribution comparison was not significant (likelihood-ratio test *p* = 0.166). Next, we compared the SNP groups with heparanase mRNA expression, plasma heparanase level, and procoagulant activity in the combined cohort. The AA and TT genotypes were associated with higher heparanase mRNA expression and procoagulant activity (Figure 2C,E). Among participants carrying the rs4693608 AA genotype, concurrent rs4364254 TT was frequent (Figure 2F). Finally, no sequence variants were identified in the amplified genomic regions encoding the heparanase procoagulant domain or the TFPI-2 inhibitory domain, or in the heparanase promoter regions analyzed with the primers described in the Materials and Methods.

**Figure 3:**
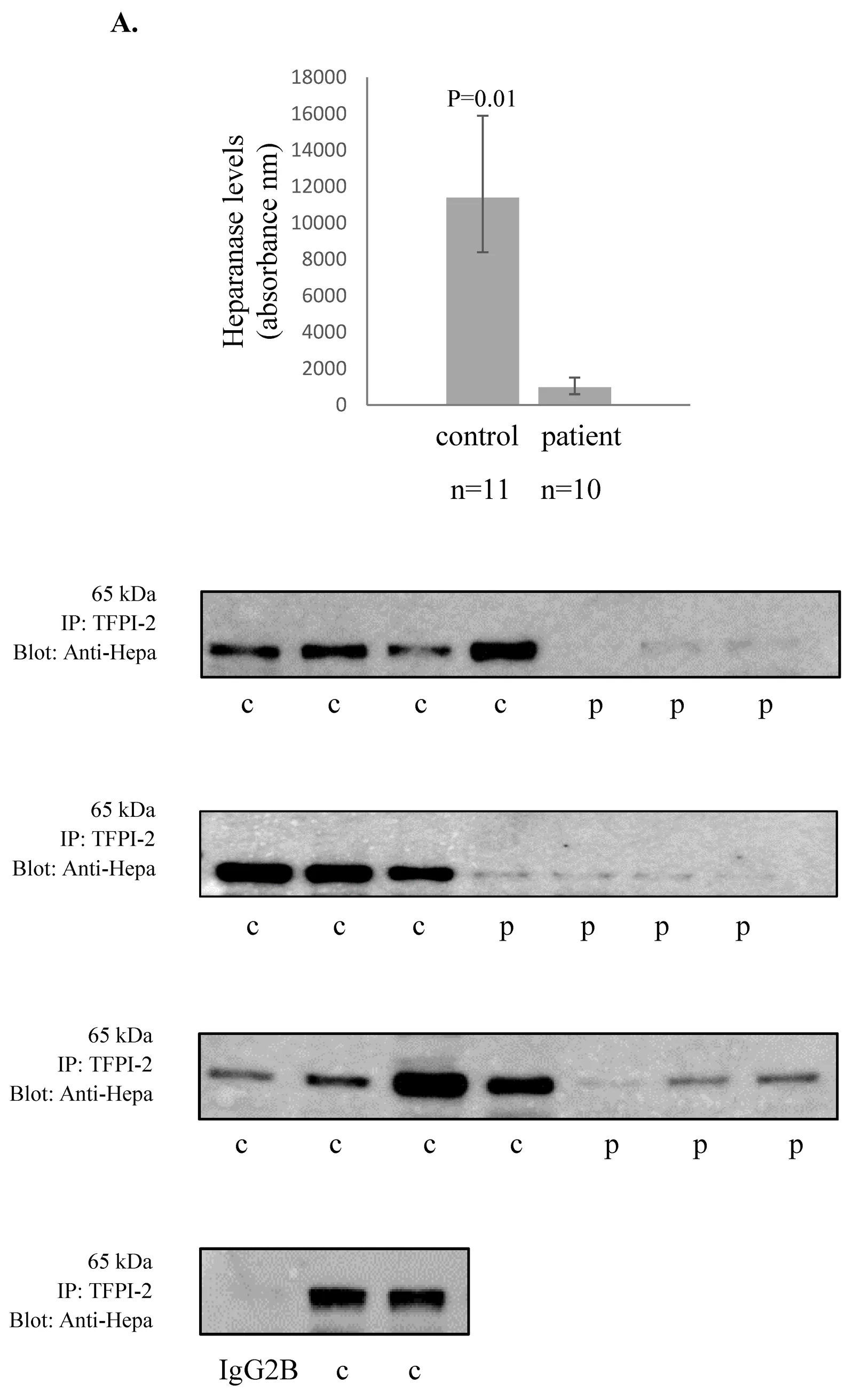

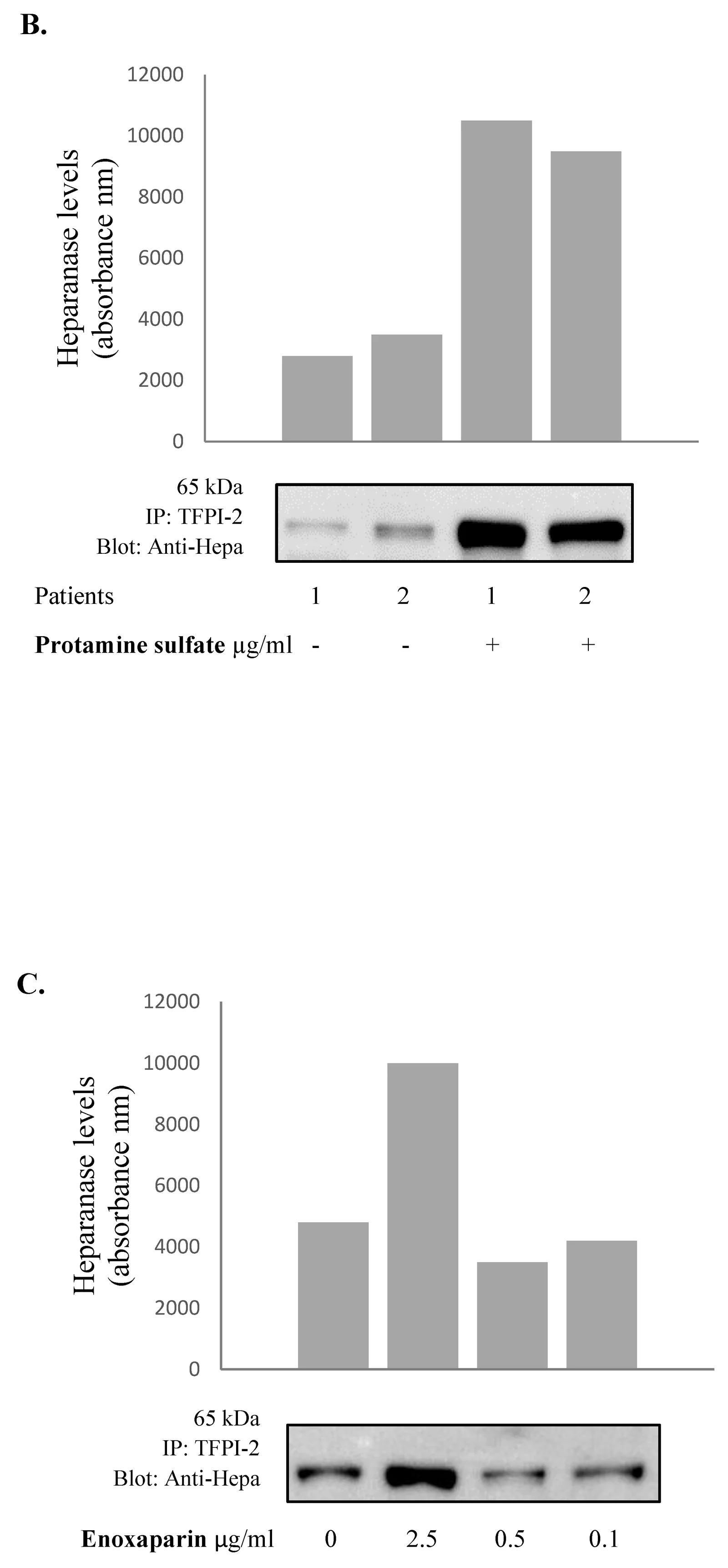

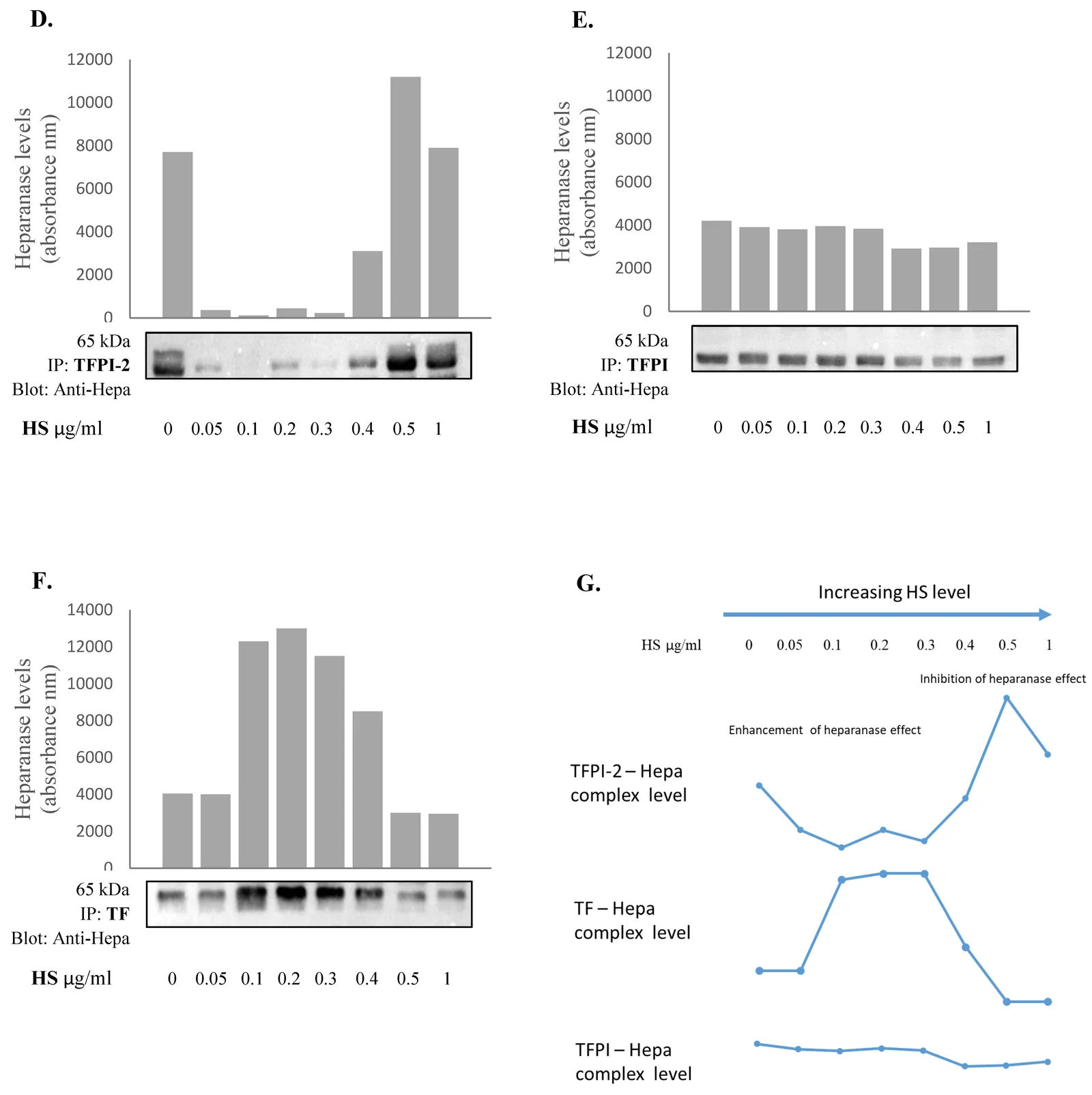
Co-immunoprecipitation (CO-IP) analysis of TFPI-2/heparanase and related complexes. (**A**) Individual plasma samples from patients (n = 10) and controls (n = 11), normalized for heparanase level by ELISA, were applied to anti-TFPI-2 columns; eluates were immunoblotted with anti-heparanase (63 IgG), and an anti-IgG2B column served as control. Mann–Whitney U test; data are shown as median and range. (**B**) Two pooled patient-plasma samples (three patients per pool; n = 6 total) were analyzed with and without protamine sulfate (5 µg/mL). (**C**) Two pooled control-plasma samples (three controls per pool; n = 6 total) were analyzed after addition of enoxaparin (0.1–2.5 µg/mL). (**D**–**F**) Pooled control plasma was analyzed after addition of HS (0–1 µg/mL) using anti-TFPI-2, anti-TF, or anti-TFPI capture columns, respectively, followed by anti-heparanase immunoblotting. (**G**) Schematic summary of the HS concentration experiment. TF—tissue factor; TFPI—tissue factor pathway inhibitor; TFPI-2—tissue factor pathway inhibitor 2; HS—heparan sulfate; ELISA—enzyme-linked immunosorbent assay.

Co-immunoprecipitation (CO-IP) was used to assess the TFPI-2/heparanase complex in individual patient and control plasma samples. Plasma samples from patients (n = 10) and controls (n = 11), randomly selected from the larger study cohort and normalized for heparanase levels, were incubated in a column conjugated with anti-TFPI-2 antibody. The eluted complex was examined by immunoblotting with anti-heparanase antibody, and an anti-IgG2B column was used as a control. TFPI-2/heparanase complex formation was lower in patients than in controls (*p* = 0.01; Figure 3A), consistent with weaker association of TFPI-2 with heparanase in the patient samples.

The protamine sulfate experiment was performed as a mechanistic ex vivo assay using pooled patient plasma rather than as a patient–control comparison. Protamine sulfate, a positively charged peptide used to reverse heparin anticoagulation, binds negatively charged heparin and related glycosaminoglycans. To test whether this could alter TFPI-2/heparanase binding, protamine sulfate (5 µg/mL) was added to each of two pooled plasma samples prepared from three randomly selected patients per pool. Samples were incubated in anti-TFPI-2 columns, and eluates were immunoblotted for heparanase. Compared with the corresponding pooled plasma without protamine, protamine-treated samples showed increased TFPI-2/heparanase complex formation (Figure 3B).

Next, we evaluated enoxaparin and HS in mechanistic ex vivo assays using pooled control plasma rather than as patient–control comparisons. Enoxaparin (0.1–2.5 µg/mL) was added to pooled plasma from three controls and incubated overnight in an anti-TFPI-2 column. At 2.5 µg/mL, TFPI-2/heparanase complex formation increased; the experiment was repeated in two pooled control samples (Figure 3C). We then examined the effects of HS on TFPI-2/heparanase, TF/heparanase, and TFPI/heparanase complexes in pooled control plasma. Heparanase was previously shown to form complexes with TF and TFPI [13], and TFPI-2 and TFPI were shown to inhibit the heparanase–TF interaction, with a stronger effect of TFPI-2 at an equivalent dose [13]. Increasing HS concentrations (0–1 µg/mL) were added to pooled plasma from three controls before application to anti-TFPI-2, anti-TF, or anti-TFPI columns; eluates were immunoblotted for heparanase. In the absence of added HS, TF/heparanase, TFPI/heparanase, and TFPI-2/heparanase complexes were detected. At intermediate HS concentrations (0–0.3 µg/mL), TFPI-2/heparanase binding decreased while TF/heparanase binding increased. At higher concentrations (0.4–1 µg/mL), the pattern was reversed; no significant effect on TFPI/heparanase binding was detected. The experiment was repeated in two pooled control samples (Figure 3D–G). Plasma HS concentrations measured in Figure 1D and exogenous HS concentrations used in the CO-IP assay were obtained in different experimental settings and therefore cannot be directly compared. Within the CO-IP experiment, increasing added HS from 0.05 to 0.1 µg/mL was accompanied by stronger TF/heparanase complex formation.

**Figure 4:**
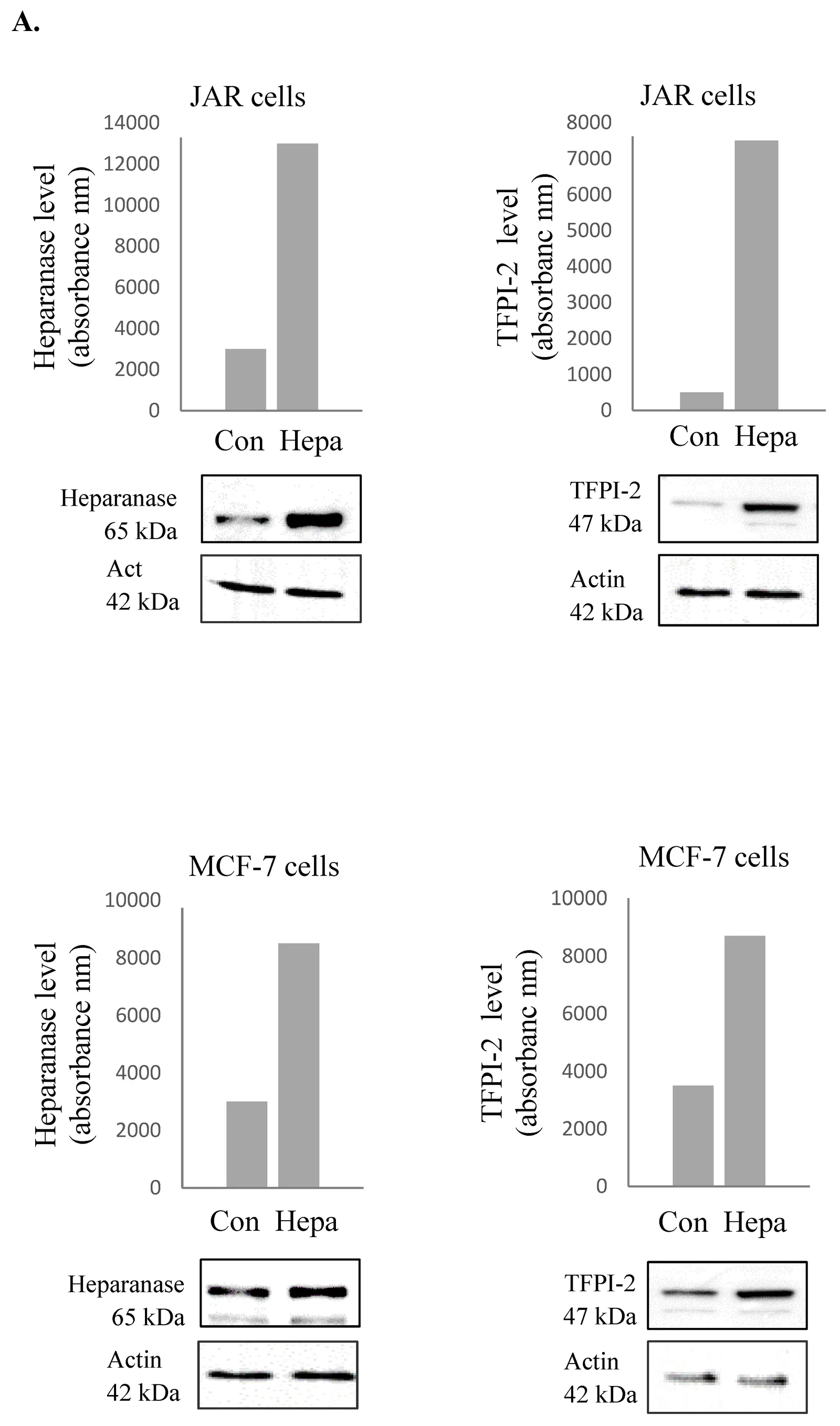

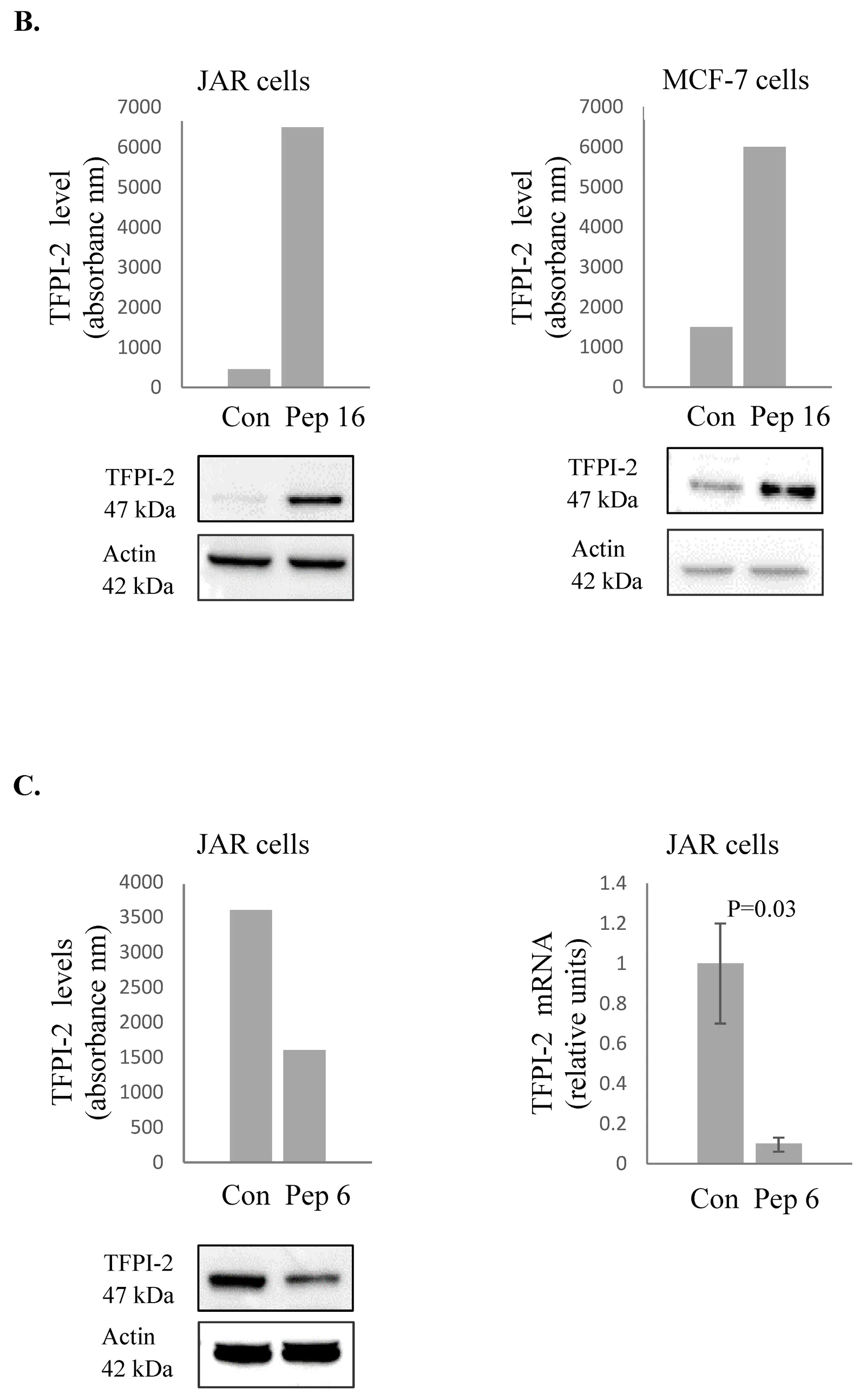
Cell-based analysis of heparanase-dependent TFPI-2 regulation. (**A**) JAR and MCF-7 cells stably transfected with heparanase (Hepa) or mock control (Con) were analyzed by immunoblotting with anti-heparanase and anti-TFPI-2; actin was used as a loading control. (**B**) JAR and MCF-7 cells were incubated overnight with heparanase-derived peptide 16 (5 µg/mL), followed by TFPI-2 immunoblotting. (**C**) JAR cells were incubated overnight with TFPI-2-derived peptide 6 (50 µg/mL), followed by TFPI-2 protein and mRNA analysis. Mann–Whitney U test; data are shown as median and range. TF—tissue factor; TFPI-2—tissue factor pathway inhibitor 2.

We then examined whether heparanase regulates TFPI-2 expression through the TF/heparanase complex. Lysates from heparanase-transfected JAR and MCF-7 cells and corresponding mock controls were immunoblotted for heparanase and TFPI-2, with actin as a loading control; increased heparanase expression was accompanied by increased TFPI-2 protein (Figure 4A). To examine the signaling mechanism, cells were incubated overnight with heparanase-derived peptide 16, which activates intracellular signaling through TF [17]. Peptide 16 increased TFPI-2 protein in JAR and MCF-7 cells (Figure 4B). To further test involvement of the TF/heparanase interaction, JAR cells were treated with TFPI-2-derived peptide 6, which inhibits this interaction [18]. Peptide 6 reduced TFPI-2 protein and mRNA levels (Figure 4C).

## 3. Discussion

In the present study, 69 women with RPL were evaluated. Although 74% of the patients had at least one thrombophilia marker and 8% had two thrombophilia markers, 26% had a normal thrombophilia work-up. Compared with controls, the patient group demonstrated higher plasma heparanase levels, procoagulant activity, heparanase mRNA expression, and HS chain levels. Importantly, blood was collected at least three months after pregnancy loss; the trimester analysis grouped women retrospectively according to the highest trimester in which a loss had occurred. Therefore, the higher heparanase level and procoagulant activity observed in the first- and second-trimester loss groups do not represent blood sampled during those trimesters and should be interpreted only as an association with the timing of the prior pregnancy loss. TFPI-2 protein and mRNA were also higher in women with pregnancy loss than in controls. In addition, the rs4693608 AA genotype was more frequent in patients and, together with the rs4364254 TT genotype, was associated with higher heparanase mRNA expression and procoagulant activity in the overall study population. Previous work by Ostrovsky et al. demonstrated that rs4693608 and rs4364254 are associated with both heparanase mRNA expression and circulating heparanase protein levels, with an inverse relationship between leukocyte mRNA expression and plasma heparanase concentration for specific genotype combinations [19]. This prior observation is relevant to the present finding that these variants are associated with heparanase-related phenotypes and supports the possibility of regulation at multiple levels.

Despite the higher TFPI-2 levels observed in patients, the interaction between TFPI-2 and heparanase was weaker than in controls. This finding raised the possibility that HS chains interfere with this interaction. Consistent with this interpretation, addition of protamine sulfate, which binds HS chains, significantly enhanced the interaction between TFPI-2 and heparanase. Notably, enoxaparin, which is structurally similar to HS, also enhanced formation of the TFPI-2/heparanase complex at a therapeutic dose in the in vitro assay. Together, these findings support a regulatory role for HS in modulating the binding partners of heparanase.

**Figure 5:**
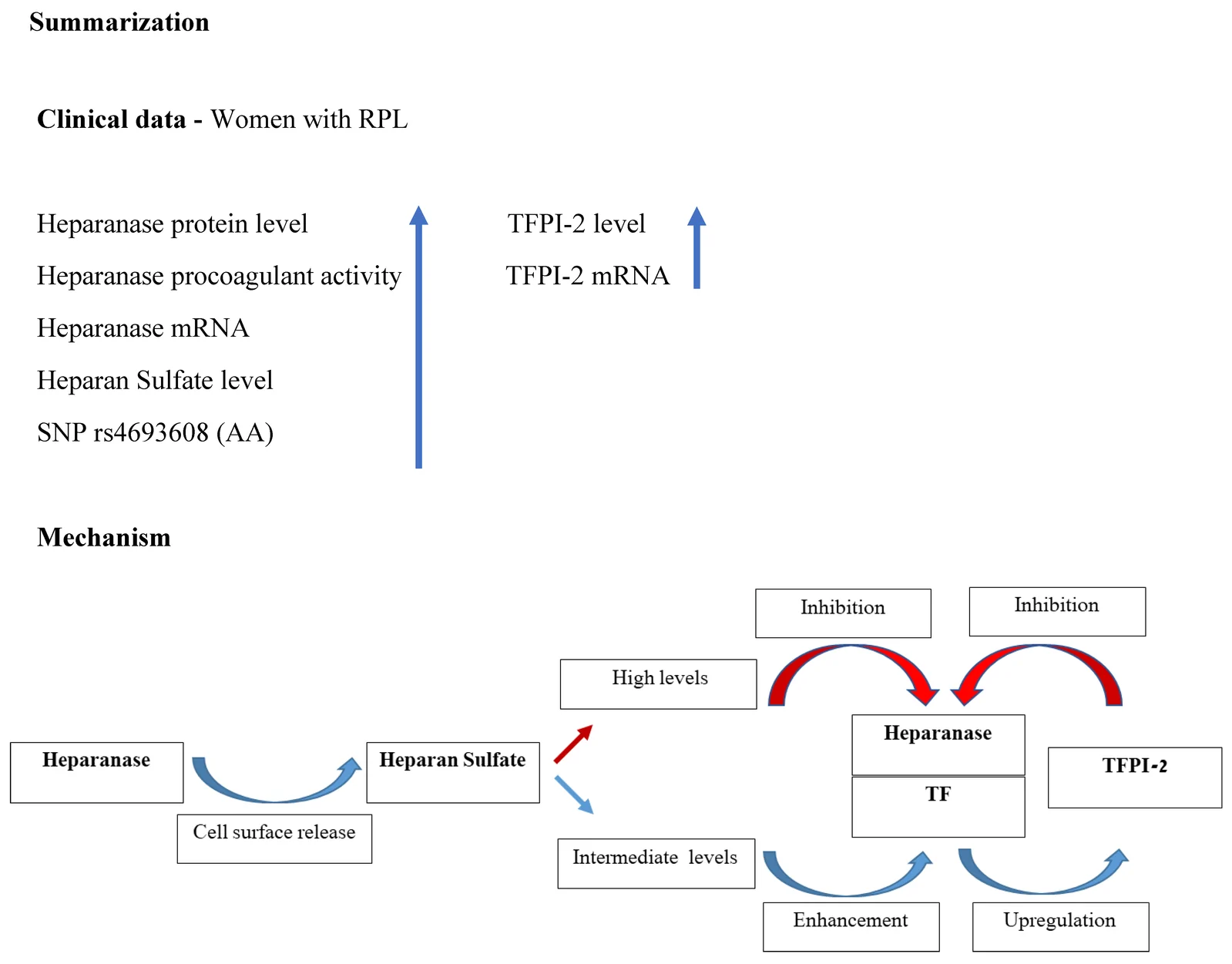
Schematic summary of the proposed heparanase/TFPI-2/HS axis in RPL. The upper portion summarizes the clinical measurements evaluated in women with RPL, including plasma heparanase level, heparanase procoagulant activity, heparanase mRNA, HS, TFPI-2 protein and mRNA, and rs4693608 genotype. The lower portion integrates the mechanistic CO-IP and cell-culture experiments: heparanase-mediated HS release, concentration-dependent HS modulation of TF/heparanase and TFPI-2/heparanase complexes, and TF/heparanase-dependent regulation of TFPI-2. Arrows indicate the direction of the proposed relationships shown in the schematic, and inhibitory arrows indicate the proposed inhibitory interactions. The diagram is a conceptual summary and does not represent additional quantitative data. RPL—recurrent pregnancy loss; TF—tissue factor; TFPI-2—tissue factor pathway inhibitor 2; HS—heparan sulfate; CO-IP—co-immunoprecipitation.

The results further suggest that HS exerts a bell-shaped effect on these interactions. At physiological concentrations, the interaction between TFPI-2 and heparanase appears to be relatively strong. At intermediate HS concentrations above the physiological range (0–0.3 µg/mL), binding between heparanase and TFPI-2 decreased, whereas binding between heparanase and TF increased. At higher HS concentrations (0.4–1 µg/mL), the interaction between heparanase and TFPI-2 was strengthened, while the interaction between heparanase and TF decreased. No significant effect of HS on binding between heparanase and TFPI was observed. Because HS levels in patients were approximately two fold higher than in controls (21 µg/mL vs. 11 µg/mL), it is plausible that elevated heparanase levels may increase HS generation, thereby shifting binding toward TF and enhancing procoagulant activity under certain conditions. At higher HS concentrations, or in the presence of therapeutic-dose heparin, TFPI-2 appears to bind heparanase more strongly, which may contribute to inhibition of heparanase activity. A summary of these findings is presented in Figure 5.

In addition to forming a complex with TFPI-2, heparanase also appeared to upregulate TFPI-2 expression. Experiments using peptides 16 and 6 suggested that this effect is mediated through the TF/heparanase complex, in line with previous evidence that this complex exerts intracellular effects [17]. These findings may indicate the existence of a regulatory loop in which heparanase promotes expression of an inhibitor that can subsequently attenuate its procoagulant function.

Current thrombophilia evaluation does not explain a substantial proportion of maternal vascular gestational pathologies [1,2,3]. In the present cohort, which consisted predominantly of women with first-trimester pregnancy losses, 26% of patients had normal thrombophilia studies. The current data therefore add support to a possible association between heparanase, TFPI-2, and RPL. TFPI-2 has previously been shown to increase throughout pregnancy, with elevated placental levels reported in pregnancies complicated by pre-eclampsia [18,20,21]. The higher prevalence of rs4693608 in patients is also notable, given its known association with increased heparanase levels. Previous studies demonstrated that heparanase can activate the coagulation system [13], and elevated heparanase levels have been reported in several clinical hypercoagulable states [1,2,22,23,24,25,26,27]. Taken together, these observations suggest that heparanase may warrant further investigation as a potential marker in RPL. However, more extensive research is needed before any consideration for routine thrombophilia screening. RPL is multifactorial, and genetic or fetal causes remain important alternative etiologies considered in current diagnostic guidelines [5]. In the present study, known fetal genetic abnormalities were excluded. Although heparanase mRNA in peripheral white blood cells and plasma heparanase protein were both increased, a subject-level correlation between these two biological compartments was not performed; therefore, concordance between mRNA and circulating protein cannot be inferred.

**Figure 6:**
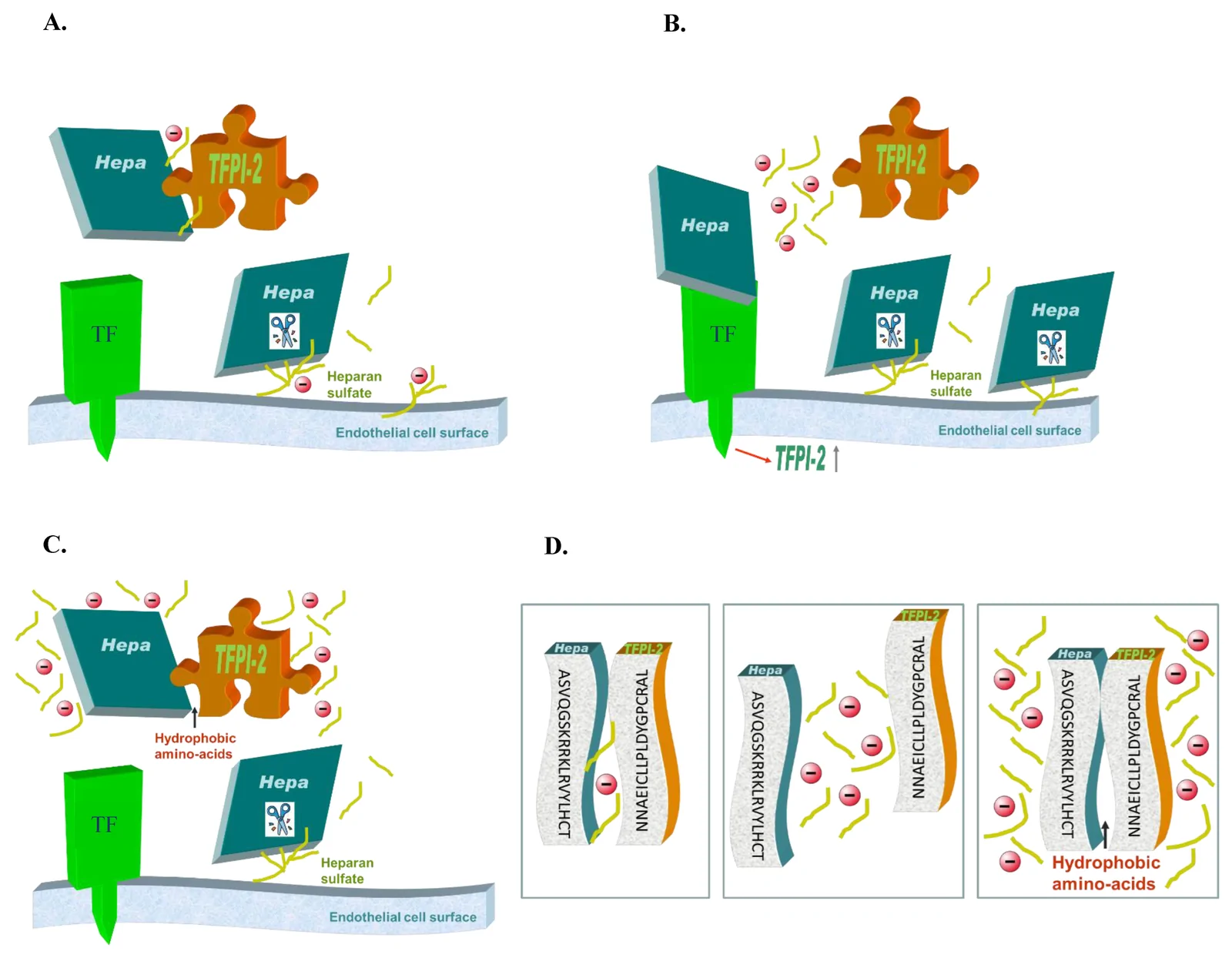
Schematic illustration of the proposed mechanism. Heparan sulfate (HS), a product of heparanase enzymatic activity, regulates heparanase function in a bell-shaped manner. At physiologic concentrations, the negative charge of HS enhances the interaction between heparanase and TFPI-2 (panel (**A**)). At higher HS levels, the increased negative charge disrupts this complex, allowing heparanase to interact with tissue factor (TF) instead. This latter interaction transduces an intracellular signal that upregulates TFPI-2 expression (panel (**B**)). At the highest HS concentrations, the strongly negative environment promotes clustering of hydrophobic residues, creating a natural hydrophobic milieu that again strengthens the interaction between heparanase and TFPI-2 (panel (**C**)). Panel (**D**): Amino acid sequences at the heparanase/TFPI-2 interaction site, as we previously described [30], are shown. Positively charged residues: lysine (K), arginine (R), and histidine (H) facilitate the interaction between the two proteins in the presence of physiologic concentrations of negatively charged HS chains. However, intermediate elevations in HS disrupt this interaction. In the strongly negative environment generated by high HS levels, hydrophobic residues: glycine (G), alanine (A), valine (V), leucine (L), isoleucine (I), and proline (P) tend to cluster together, forming a natural hydrophobic microenvironment.

Previous studies showed that TFPI-2 and TFPI inhibit the procoagulant effect of heparanase by interfering with its interaction with TF [13]. Heparanase was also shown to upregulate TF [11] and TFPI [12], and the present results suggest that TFPI-2 may be similarly upregulated. Such coordinated upregulation may help explain why this group of related proteins is often elevated in clinical settings associated with increased heparanase activity [1,28,29]. An unresolved question, however, is why the interaction between heparanase and TF is not fully neutralized in the presence of TFPI and TFPI-2. The current data suggest that circulating HS chains may contribute to this regulation. In women with pregnancy loss, the weaker interaction between TFPI-2 and heparanase was associated with higher heparanase and HS levels. Neutralization of HS by protamine sulfate enhanced the TFPI-2/heparanase interaction, and therapeutic-equivalent enoxaparin had a similar effect. These observations support the idea that HS, a product of heparanase enzymatic activity, may regulate heparanase non-enzymatic activity by altering its preferential interaction with TF or TFPI-2. As HS levels rise, interaction with TF appears to predominate at intermediate concentrations, whereas at higher concentrations interaction with TFPI-2 becomes dominant again. A schematic illustration of this proposed mechanism is presented in Figure 6.

This model may also have implications for the mechanism of action of heparins in RPL. The effect of therapeutic-dose enoxaparin in enhancing the TFPI-2/heparanase interaction resembles the effect observed with high HS concentrations. In addition, unfractionated heparin was previously shown to inhibit the interaction between heparanase and TF [13]. These findings raise the possibility that, beyond their established anticoagulant effects, heparins may modulate the heparanase–TF/TFPI-2 axis. Previous work showed that, in high-risk pregnancies treated with prophylactic low-molecular-weight heparin, anti-Xa-guided monitoring was associated with better pregnancy outcomes than weight-adjusted dosing [31]. A five-fold lower dose of enoxaparin compared with the therapeutic dose (0.5 µg/mL vs. 2.5 µg/mL) does not reach the recommended fixed prophylactic dose of enoxaparin and may explain the lack of enhancement of the TFPI-2/heparanase complex. To accurately determine the dose-dependent effect of enoxaparin on this complex, increasing doses of enoxaparin should be administered subcutaneously in a mouse model in future studies.

Study limitations: The combination of clinical samples with mechanistic assays is a strength of this study; however, the clinical cohort size remains modest, particularly for genotype-based and subgroup analyses. Although most patients experienced first-trimester pregnancy loss (75%), the patient group remained heterogeneous, which may confound interpretation. In addition, the thrombophilia work-up was performed only in the study group, limiting direct comparison with controls. Because heparanase is closely linked to coagulation, the absence of thrombophilia testing in controls may have influenced between-group comparisons. According to recent literature, among thrombophilia-related markers, only lupus anticoagulant, anti-cardiolipin antibodies, and anti-β2-glycoprotein antibodies are considered clinically relevant in recurrent pregnancy loss [5,32,33]. Other inherited thrombophilias are not routinely considered part of the pathology of pregnancy loss. In our cohort, only seven patients had positive antiphospholipid markers, while 18 had a normal thrombophilia work-up; therefore, these groups were too small for meaningful comparison. Several mechanistic experiments relied on pooled plasma or small sample numbers, thereby weakening the strength of some conclusions. In addition, the exclusion criteria included known abnormalities in kidney or liver function tests. Although occult kidney or liver abnormalities are uncommon at a mean age of 31–32 years, this possibility cannot be fully excluded. Because plasma testing was performed outside active gestation, these findings should be interpreted as indirect surrogate markers of pregnancy-related pathologies rather than direct evidence of the early placental environment during spiral artery remodeling. Further studies evaluating the heparanase/TFPI-2/HS axis throughout pregnancy are needed to better support the involvement of this axis in normal and pathological pregnancies. Because blood samples were collected at least three months after pregnancy loss, the circulating measurements may not fully reflect the maternal–placental environment at the time of the loss, and the observed clinical associations should not be interpreted as causal. In placenta accreta spectrum disorders, recent single-cell and spatial transcriptomic analysis demonstrated alterations in trophoblast populations at invasion sites and in the local myometrial immune microenvironment, further emphasizing the importance of the maternal–fetal tissue context [34]. Before heparanase can be considered a clinical biomarker for RPL, its performance should be validated in independent, preferably prospective pregnancy cohorts; the use of independent cohorts has also been emphasized in recent pregnancy-prediction research [35]. The plasma TFPI-2 Western blot was performed on a small subset and should therefore be considered exploratory and interpreted together with the independent TFPI-2 mRNA analysis. Equal volumes of plasma were loaded for the plasma immunoblots; no single universally accepted invariant housekeeping protein exists for plasma, in which abundant proteins also show interindividual biological variation.

## 4. Materials and Methods

**Study group:** This study was approved by the institutional Ethic Committee on Human Research at Rambam Medical Center no. 0068-20-RMB. The study group included 69 women with pregnancy losses and 47 controls enrolled over a 3-year period, between January 2020 and December 2022, at the Rambam Medical Center. Inclusion criteria were defined as women with recurrent pregnancy loss (RPL) that included 3 or more first-trimester miscarriages, two or more second-trimester miscarriages or one third-trimester fetal loss. The control group included women with two or more normal deliveries and no history of pregnancy vascular complications (RPL, pre-eclampsia, preterm birth < 37 weeks, small for gestational age < 5th percentile, placental abruption). Exclusion criteria included age under 18 years, hormonal therapy or pregnancy within the 3-month period prior to recruitment, evidence of genetic abnormalities in the fetus, evidence of infection in the fetus or mother during pregnancy, concomitant medications other than supplement minerals and vitamins, acute or chronic illness, and known kidney or liver test abnormalities.

**Reagents and antibodies:** A single-chain GS3 heparanase gene construct, comprising the 8 and 50 kDa heparanase subunits (8 + 50) was purified from the conditioned medium of baculovirus-infected cells. GS3 heparanase was assayed for the presence of bacterial endotoxin (Biological Industries, Beit Haemek, Israel), using the gel-clot technique (limulus amebocyte lysate—LAL test) and was found to contain <10 pg/mL of endotoxin [11]. Polyclonal antibody 63 was raised in rabbits against the entire 65 kDa heparanase precursor isolated from the conditioned medium of heparanase-transfected HEK-293 cells. The antibody was affinity-purified on immobilized bacterially expressed 50 kDa heparanase glutathione-S-transferase (GST) fusion protein [36]. Monoclonal anti-heparanase antibody 4B11 was generated by immunizing BALB/C mice with the entire 65 kDa heparanase protein [36]. Recombinant human factor VIIa and plasma-derived human factor X were purchased from Sekisui Diagnostics, Inc. (Stamford, CT, USA). Recombinant human TFPI-2 was purchased from R&D systems (Minneapolis, MN, USA). All coagulation factors were dissolved in double-distilled water. Chromogenic substrate to factor Xa (I.D. 222, solubility: Tris buffer, pH −8.4) was purchased from Sekisui Diagnostics (Stamford, CT, USA). Bovine factor Xa and was obtained from Sigma-Aldrich Corp (St. Louis, MO, USA). Polyclonal anti-human TF and polyclonal anti-human TFPI antibodies were purchased from Santa Cruz (Santa Cruz, CA, USA). Polyclonal anti-TFPI-2 was purchased from Bioss (Woburn, MA, USA). Monoclonal anti-Actin was purchased from Santa Cruz (Santa Cruz, CA, USA). The amino acid sequence of peptide 16AC derived from the procoagulant domain of heparanase was amid-GSKRRKLRVYLHCT-acetyl (amid and acetyl residues prevent peptide degradation and enhance activity). The amino acids sequence of peptide 6 derived from TFPI-2 that inhibited the interaction between TF and heparanase was AEICLLPLDYGPCR, as previously described [30,37].

**Blood and plasma samples:** Written informed consent was obtained from all participants. Human peripheral blood was freshly collected in 10 mL tubes containing 3.2% sodium citrate as anticoagulant. The tubes were centrifuged at 1500× *g* for 15 min at room temperature to obtain platelet-poor plasma and the upper cellular fraction (buffy-coat fraction) containing white blood cells. Samples were stored at −80 °C.

**Heparanase procoagulant activity assay**: Heparanase procoagulant activity was quantified by a factor Xa generation assay; all concentrations listed below are the final assay concentrations. Twenty-five microliters of the plasma, recombinant human factor VII (0.04 µM), and plasma-derived human factor X (1.4 µM) were incubated in a 50 µL assay buffer [0.06 M Tris, 0.04 M NaCl, 2 mM CaCl2, 0.04% (*w*/*v*) bovine serum albumin (BSA), pH 8.4] to a total volume of 125 µL in a 96-well sterile plate. After 15 min at 37 °C, chromogenic substrate was added to factor Xa (1 mM). The levels of Xa activity were determined using an ELISA plate reader at an absorbance of 405–490 nm (PowerWave XS; BioTek, VT, USA). Heparins were shown to abrogate the TF/heparanase complex, so in parallel, the same assay was performed except that fondaparinux (2.5 µg/mL) was added to the assay buffer [13]. Bovine factor Xa diluted in the assay buffer was used to generate a standard curve. The subtraction of the first assay result from the second assay result determined heparanase procoagulant activity.

**Heparanase Enzyme-Linked Immunoassay (ELISA).** Wells of microtiter plates were coated (18 h, 4 °C) with 2 µg/mL of anti-heparanase monoclonal antibody 4B11 in 50 µL coating buffer (0.05 M Na_2_CO_3_, 0.05 M NaHCO_3_, pH 9.6). The plate was covered with adhesive plastic and incubated overnight at 4 °C. The next day, wells were blocked with 2% BSA in PBS for 1 h at room temperature 23 °C. Diluted samples (100 µL) were loaded in duplicates and incubated for 2 h at room temperature, followed by the addition of 100 µL anti-heparanase polyclonal antibody 63 IgG (1 µg/mL) for an additional period of 2 h at room temperature. HRP-conjugated goat anti-rabbit IgG (1:20,000) in blocking buffer was added (1 h at room temperature) and the reaction was visualized by the addition of 100 µL chromogenic substrate (TMB; MP Biomedicals, Germany) for 15 min. The reaction was stopped with 100 µL H_2_SO_4_ and the absorbance of the samples was measured at the wavelength of 450–630 nm using an ELISA plate reader (PowerWave XS, Bio-Tek Instruments, Winooski, VT, USA). Plates were washed four times with washing buffer (PBS, pH 7.4 containing 0.1% (*v*/*v*) Tween 20) after each step. As a reference for quantification, a standard curve was established by serial dilutions of recombinant 8 + 50 GS3 heparanase (390 pg/mL–25 ng/mL). The assay reveals the active heparanase almost exclusively and only poorly reveals the full-length heparanase (65 kDa). This ELISA method was performed as previously described [38].

**Heparan sulfate chain quantitation chromogenic assay:** Heparan sulfate (HS) levels were measured in the following way: 16 mg of dimethylmethylene blue (DMMB, Sigma-Aldrich) were dissolved in 1 L of double-distilled water containing 3 g glycine, 1.6 g NaCl and 95 mL of 0.1 M acetic acid. The solution was filtered using Whattman 3 MM. A standard curve was prepared using HS derived from bovine kidney (Sigma-Aldrich, H7640). Although HS differs by species and tissue source in chain length, disaccharide composition, and sulfation pattern, bovine kidney HS is often used experimentally as a commercially available HS model [39]. To 20 µL of a sample, 5 µL of protamine sulfate (10 mg/mL, Fresenius Kabi, Toronto, Canada) was added followed by the addition of 20 µL of a sample and 5 µL of UPW (ultra-pure water). To each of these samples, 200 µL of DMMB solution was added. Absorbance was immediately read using a plate reader at 525 nm. Each sample was evaluated with and without protamine sulfate and the subtraction of the first assay result from the second assay result determined the HS level in a sample. This HS quantitation method was performed as previously described [40].

**RNA isolation and cDNA preparation:** Total RNA was extracted from the buffy-coat fraction containing white blood cells by using Trizol Reagent (Molecular Research Center, Inc. [MRC], Cincinnati, OH, USA). Next, 1 mL Trizol was added to 200 µL of the buffy-coat fraction, which was incubated for 5 min on ice. Then, 0.2 mL chloroform was added to each tube, shaken vigorously for 15 s and incubated for 10 min at on ice. After the samples were centrifuged at 12,000× *g* for 15 min at 4 °C, the aqueous phase was transferred to a fresh tube. The RNA from the aqueous phase was precipitated by adding 500 µL isopropanol for 10 min on ice. Next, the samples were centrifuged at 12,000× *g* for 8 min at 4 °C. After that, the supernatant was removed and the RNA pellet was washed with 75% ethanol and centrifuged at 7500× *g* for 5 min at 4 °C. In the end, the RNA pellet was briefly air-dried, dissolved in DEPC (Sigma, USA)-treated RNases free water. The concentration and purity of the samples were measured in terms of (OD_260_/OD_280_) by using a spectrophotometer (Epoch BioTek, Santa Clara, CA, USA). RNA (1 µg) was resolved by 1% agarose gel electrophoresis and was visualized by Gelstar nucleic acid gel stain at ImageQuant las 4000 system. Then, 1 µg of RNA was added to a new tube to synthesize the first-strand complementary DNA (cDNA) by using qScript cDNA synthesis kit (Quanta biosciences, USA). The tubes were placed in PCR machine in a thermal cycler program as follows: 22 °C for 5 min, 42 °C for 30 min and 85 °C for 5 min.

**Real-time PCR**: Quantitative real-time polymerase chain reaction (qRT-PCR) was conducted to detect the relative mRNA levels. RT-PCR was performed using the SYBR green FastMix ROX (Quanta biosciences, USA) on the QuantStudio3 Real Time PCR System (applied-biosystems, USA). Glyceraldehyde-3-phosphate dehydrogenase (GAPDH) was used as an endogenous control. Then, 40 ng of cDNA was added for each well; all samples were tested in triplicate. The following conditions were applied for TFPI-2, heparanase and GAPDH (control gene) PCR amplification: 50 °C for 2 min, 95 °C for 10 min, 40 cycles of 95 °C for 15 s and of 60 °C for 1 min. The results were calculated using 2^(−ΔΔCt)^ equation.

**DNA extraction from blood:** Extracting DNA relies on separation of the white blood cells (which contain DNA) from the whole blood cell. DNA was isolated from 200 µL of white blood cells from the buffy-coat fraction collected after plasma removal by using the High Pure PCR Template Preparation Kit (Roche Diagnostics GmbH, Mannheim, Germany) according to the manufacturer’s protocol via the following procedure: White blood cells were thoroughly vortexed and RNase treated for 15 min at 37 °C. Moreover, fragments of proteins, membranes and organelles were subsequently removed from the sample. The DNA pellet was precipitated with ethanol, briefly dried and resuspended in 30 µL of elution buffer. The concentration and purity of the samples were measured in terms of (OD_260_/OD_280_/OD_230_) by using a spectrophotometer (Epoch BioTek, USA).

**Primers design:** Each PCR reaction contained one forward/reverse primer pair specific to a single target region. After acquisition of the target genes’ DNA sequences from the NCBI Nucleotide database (http://www.ncbi.nlm.nih.gov/) accessed on 19.1.2021, primers were designed for each gene using the Primer 3 online server (https://primer3.org/input.htm, accessed on 7 September 2026). Primers were acquired from Syntezza Bioscience Ltd. (Jerusalem, Israel) and their characteristics appear below. The primers used to evaluate the DNA sequence were as follows: heparanase procoagulant domain primers F-TGTAGGATAACTATTTGCTTGC, R-TATAGACACATGCAACAGGG, TFPI-2 anticoagulant domain F-GGGACTCGCTCCCAAGTTT, R-CCAGGCTAAAACTTCCTGTAGA, SNP rs4364254 F-CCACCATGAATCTGAGAGCA, R-TTTGGCTTTGAGCTTTGCTT, SNP rs4693608 F-TGAAGAAAACATAGTGCAGTTCTC, R-AAGCTATCCATCTGCCTTGG, heparanase promoter 1 F-CTTAGCTTTTAAGTTGCTT, R-AGAGTCTAATAAATATTGGTTG, heparanase promoter 2 F-AATCTCTGTTTTGGTGGAGCATATAA, R-CTCCAATGTCTGGAACTCAGGAC, heparanase promoter 3 F-TCCTGAGTTCCAGACATTGGAG, and R-CACTTGAAGGGTGCGGAGA.

**PCR technique:** PCR amplification of DNA was done by using AccuStart GelTrack PCR SuperMix, acquired from Quanta Biosciences (Tel-Aviv, Israel) according to the manufacturer’s instructions. For DNA amplification, we put the following substances in a sterile eppendorf: 6 µL of DNA template (240 ng\µl), 12.5 µL of 2x PCR Supermix (which containing dNTP mix, Taq DNA polymerase, MgCl_2_, reaction buffer and gel-loading dye), and 2 µL (500 nM) primers. Then, 4.5 µL of RNAase free water was added to reach a total reaction volume of 25 µL. The reaction was performed in the thermal cycler apparatus of Tamar Ltd. (Mevaseret Zion, Israel) using a program consisting of two steps: denaturation of the double-stranded DNA in 94 °C for 60 s followed by 25 cycles of 94 °C for 30 s (allowing full activation of Taq polymerase), different annealing temperatures according to the TM of each gene, and 72 °C for 60 s. The specificity of primers and PCR products were analyzed on 3% agarose gel.

**Sanger sequencing of PCR product:** In order to dispose of unincorporated primers and degraded nucleotides, PCR products were cleaned with the EXO-Sap kit (containing Exonuclease I and Alkaline Phosphatase enzymes) of Tamar Ltd. (Mevaseret Zion, Israel) according to the manufacturer’s instructions. After clean-up, the DNA sequence was analyzed with the Sanger sequencing method (in the Biomedical Core Facility of Medicine, Technion, Israel) which is based on the selective incorporation of chain-terminating dideoxynucleotides.

**SDS–polyacrylamide gel electrophoresis (PAGE) and immunoblot analysis.** Samples for Western blot analysis were randomly selected from the larger study cohort. For plasma Western blotting, an equal volume of plasma from each participant was loaded in each lane. This volume-based approach was selected because plasma does not have a universally accepted invariant housekeeping protein and the concentrations of individual plasma proteins, as well as total plasma protein, can vary biologically between individuals. Equal-volume plasma loading has also been used for plasma immunoblotting in published studies [41,42]. For cell lysates, actin was used as a loading control, as shown in Figure 4. Proteins were subjected to 10% SDS-PAGE and transferred to polyvinylidene fluoride membrane (BioRad, Maylands, CA, USA). The membrane was probed with the appropriate antibody followed by horseradish peroxidase-conjugated secondary antibody (Jackson ImmunoResearch, West Grove, PA, USA) and chemiluminescence substrate (Pierce, Rockford, IL, USA). Rabbit anti-heparanase (63 IgG) diluted to 1/2000 and Rabbit anti-TFPI2 diluted to 1/1000 were used, as previously described [11].

**Co-immunoprecipitation**: The interaction between TFPI-2, TFPI, TF, and heparanase was analyzed by co-immunoprecipitation (CO-IP). The Pierce Co-IP kit in which the antibody is coupled to gel support was employed according to the manufacturer’s instructions (Thermo Fisher Scientific, Waltham, MA USA). Briefly, coupling gel was washed with coupling buffer and polyclonal anti-TF, polyclonal anti-TFPI or polyclonal anti-TFPI-2 100 µg and 3 µL of 5 M sodium cyanoborohydride was added to the gel support and incubated at 4 °C overnight. The gel was then washed with quenching buffer and incubated with the quenching buffer and sodium cyanoborohydride at 20 °C for 30 min. Next, the gel was washed four times with wash solution, once with elution buffer, and twice with coupling buffer. Plasmas which express heparanase 65 kDa were added to the gel and incubated for 2 h at room temperature. The gel was washed four times with coupling buffer and bound proteins were eluted with elution buffer (pH 2.5), neutralised by 1 M Tris-HCl, pH 9.5, and subjected to immunoblot analysis. Monoclonal anti-heparanase antibody 4B11 was used to detect the respective coupled protein. An irrelevant anti-IgG2B monoclonal antibody (100 µg) was used as control.

**Cell cultures and transfection:** JAR (human choriocarcinoma) and MCF-7 (human breast carcinoma) cell lines were purchased from the American Type Culture Collection and cultured in 37 °C, 5% CO2. JAR cell line was grown in DMEM (Biological Industries, Beit Haemek, Israel) and supplemented with 10% fetal bovine serum (FBS), 1% L-glutamine and 1% penicillin–streptomycin amphotericin (rich medium). MCF-7 cell line was grown in RPMI medium (Biological Industries, Beit Haemek, Israel) and supplemented with 10% FBS, 1% L-glutamine and 1% penicillin, streptomycin and amphotericin. JAR and MCF-7 cells were stably transfected with the human heparanase cDNA cloned into the pSecTag2 vector (Invitrogen, Carlsbad, CA, USA), using the FuGENE 6 reagent, according to the manufacturer’s instructions (Roche Applied Science, Indianapolis, IL, USA). Transfection proceeded for 48 h, followed by selection with Zeocin for 2 weeks. Stable transfectant pools were further expanded and analyzed.

**Statistical analysis:** Data were evaluated using the SPSS software for Windows version 27 (SPSS Inc., Chicago, IL, USA). Statistics were calculated by the parametric unpaired *T*-test, Mann–Whitney U test or Chi-square test, or Fisher’s exact test, as designated. Values were reported as the mean and SD. The significance levels were set at *p* < 0.05. For the additional genetic analysis, genotype distributions were compared by the Chi-square test when expected cell counts were adequate; allele frequencies and dominant/recessive models were evaluated with two-sided Fisher’s exact tests and odds ratios with 95% confidence intervals. Hardy–Weinberg equilibrium was assessed in controls. Two-locus haplotype frequencies were estimated from unphased rs4693608/rs4364254 genotypes by expectation-maximization, and the global difference in haplotype distributions between patients and controls was assessed by a likelihood-ratio test. These genetic analyses were considered exploratory because of the sample size.

## 5. Conclusions

The present findings support a possible role for heparanase and TFPI-2 in the pathophysiology of RPL. Elevated heparanase levels, procoagulant activity, mRNA expression, HS levels, and the higher prevalence of specific SNPs may together point to a heparanase-related thrombophilic profile in a subset of women with RPL. The data also suggest that HS chains may play a physiological regulatory role by modulating the interactions of heparanase with TF and TFPI-2, and that heparins may influence this balance because of their structural similarity to HS. These observations should be considered hypothesis-generating and warrant further validation in larger clinical and mechanistic studies.

## Figures and Tables

**Table 1:** Thrombophilia work-up results of the study group.

	Study Group n = 69 (%)
Normal work-up	18 (26%)
Lupus anticoagulant	4 (6%)
Anti-cardiolipin	5 (7%)
Anti-β2glycoprotein	5 (7%)
Prothrombin mutation Hetero	10 (14%)
Prothrombin mutation Homo	0
Factor V Leiden Hetero	14 (20%)
Factor V Leiden Homo	1 (1%)
Factor VIII (>200%)	7 (10%)
Homocysteine	3 (4%)
Protein C	2 (3%)
Protein S	6 (9%)
Anti-thrombin	0

## Data Availability

For original data, please contact y_nadir@rambam.health.gov.il.
